# Effects of retractor application on cuff pressure and vocal cord function in patients undergoing anterior cervical discectomy and fusion

**DOI:** 10.4103/0019-5049.68370

**Published:** 2010

**Authors:** Rakesh Garg, Girija P Rath, Parmod K Bithal, Hemanshu Prabhakar, Manish K Marda

**Affiliations:** Department of Neuroanaesthesiology, All India Institute of Medical Sciences, New Delhi, India

**Keywords:** Anterior cervical discectomy and fusion, cervical retraction, cuff pressure, vocal cord function supraclavicular block

## Abstract

Anterior cervical discectomy and fusion is a commonly performed procedure for prolapse of cervical intervertebral disc. It involves retraction of soft tissue of neck for adequate exposure of anterior spinal canal. Increased cuff pressure with retractor application may affect the postoperative vocal cord function. Cuff pressures of tracheal tube were measured continuously in 37 patients using air-filled pressure transducer connected to the pilot balloon. Changes of pressure from baseline values were noted after application of cervical retractor. At the end of procedure, vocal cord movement was observed using fibreoptic bronchoscope. Significant increase in cuff pressure (168% of baseline values) and airway pressure of tracheal tube during cervical retraction was observed. The vocal cord function was assessed using fibreoptic laryngoscope. One patient developed right vocal cord palsy (2.7%) and two patients had postoperative hoarseness of voice (5.4%). All these complications improved over a period of time. It is suggested that the cuff of tracheal tube should be inflated to achieve ‘just seal’, with adequate cuff pressure monitoring. Intermittent release of cervical retraction may help to prevent laryngeal morbidities.

## INTRODUCTION

Anterior cervical discectomy and fusion (ACDF) is a commonly performed procedure for prolapse of intervertebral disc. ACDF involves anterior neck dissection, retraction of the soft tissue that gives exposure to the anterior vertebral column. Application of cervical retractors may increase the cuff pressure of endotracheal tube (ETT), which may lead to laryngo-tracheal morbidities like hoarseness of voice, sore throat, dysphagia, and vocal cord palsy.[1] Incidence of tracheal ischaemia after ACDF has been reported to be 2-44%.[1] The relation between retractor application and change in cuff pressure of ETT has rarely been mentioned in the literature.[2,3] Hence, this study was designed to find out changes in cuff and airway pressures with retractor application during ACDF, with a secondary objective to assess vocal cord function after extubation.

## METHODS

**Figure 1:**
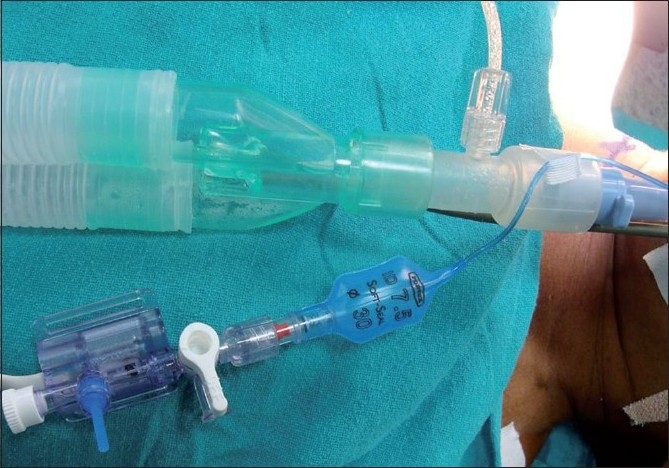
Air-filled pressure transducer attached to pilot balloon of tracheal tube for continuous cuff pressure measurement

This prospective, within subject type, observational study was carried out after obtaining approval of Institute Ethics Committee. Informed written consent was taken from patients belonging to ASA physical status I and II, who underwent elective ACDF over a period of one year. Patients belonging to age group of 18-65 years were included in the study. Patients having short neck, anatomical deformity or history of previous surgery at neck or cervical spine, obesity, and history of smoking were excluded from the study. During preanaesthetic visit the patients were explained about the procedure and the nasal use of fibreoptic bronchoscope (FOB) at the time of tracheal extubation. In the operating room, routine monitors such as ECG, noninvasive blood pressure, pulse oximeter, and sensor for bispectral index (BIS) monitoring were attached. Anaesthesia was induced with intravenous fentanyl (2 μg/kg), propofol (2–2.5 mg/kg), and tracheal intubation was facilitated with rocuronium (1mg/kg). Fiberoptic bronchoscope guided orotracheal intubation was achieved with appropriate size tracheal tube (7.5mm ID for female and 8.5 ID for male patients; Portex® Tracheal tube, Smiths Medical International Ltd, USA). Cuff was inflated with air to a point at which there was no leak on auscultation at the suprasternal area, at an inspiratory pressure of 20 cm H_2_O. The pilot balloon of ETT was attached to an air-filled pressure transducer for continuous cuff pressure measurement [Figure 1]. The cuff pressure was measured at end-inspiration. Anaesthesia was maintained with 40% oxygen in air and propofol infusion titrated to maintain bispectral index (BIS) value of 40-60. Intermittent bolus of rocuronium was administered with neuromuscular monitoring. The supplemental analgesia was provided with fentanyl (1 μg/kg) every hourly. Parameters like cuff pressure, mean and peak airway pressures, heart rate (HR), and mean arterial pressure (MAP) were recorded at 5 min after tracheal intubation (baseline), just before and 1 min after Cloward® retractor application, and immediately after retractor was removed. At the end of surgery, residual neuromuscular blockade was reversed, and fibreoptic bronchoscope was inserted through either nostril. After achieving a train-of-four count (TOF) of >90%, the ETT was withdrawn while observing for mobility of vocal cords. Any abnormality in the form of oedema or bleeding in the supraglottic area was also noted. Postoperatively, the patients were observed for hoarseness of voice and dysphagia for 24 hours. Patients having any such morbidity were followed-up for subsequent 3 months.

The study parameters at different time interval were statistically compared using generalized estimating equation. The *P* value <0.05 was considered as significant. Data were presented as mean (SD), range, number or proportion.

## RESULTS

A total of 37 patients of ASA physical status I and II (19 and 18 in number, respectively) were enrolled in this study. The mean age was 48.6 ± 9.8 years (Range: 27–70 years) with male/female ratio of 33:4. The mean weight was 61.6 ± 12.2 kg (Range: 45-90 kg). There was a significant increase in cuff pressure from the baseline values, after application of cervical retractors (*P* =0.001) [Table 1]. Moreover, in 17 patients, the increase (cuff pressure) was more than 20 mmHg after retractor application. Simultaneously, there was an increase in mean and peak airway pressures from baseline values (*P* =0.001). The mean duration of retractor application was 64.9 ± 27.7 min (Range: 30-130 min). One/two/three vertebral disc level procedures were performed in 26, 9 and 2 patients, respectively. Two patients developed hoarseness of voice (5.4%) among which one patient also had right vocal cord palsy (2.7%), as observed on FOB. The complications gradually improved over next 30 days. The cuff pressure increased from 15 to 26 mmHg after application of retractor (*P* =0.001) in the patient who developed both vocal cord palsy and hoarseness while it increased from 13–33 mmHg (*P* =0.001) in the patient who developed hoarseness only.

**Table 1:** Cuff pressure, airway pressures and haemodynamics at various stages (mean ± SD, range)

Parameters	Baseline	Before application of retractor	1 min after application of retractor	After removal of retractor
Cuff pressure (mmHg)	11.8 ± 3.8 (4-18)	13.8 ± 5.2 (5-28)(*P* = 0.001)	19.9 ± 7.9 (7-40)(*P* = 0.001)	13.4 ± 4.8 (5-26)(*P* = 0.001)
Mean airway pressure (mmHg)	5.4 ± 1.4 (3-10)	5.7 ± 1.4 (3-10)(*P* = 0.03)	5.9 ± 1.7 (3-11)(*P* = 0.001)	5.7 ± 1.4 (3-10)(*P* = 0.03)
Peak airway pressure (mmHg)	14.9 ± 3.3 (9-24)	15.7 ± 3.7 (8-25)(*P* = 0.001)	16.1 ± 3.7 (10-25)*P* = 0.001)	15.8 ± 4.1 (8-26)(*P* = 0.02)
Heart rate (Beats/min)	82.8 ± 15.6 (51-127)	79 ± 15.3 (54 – 114)(*P* = 0.01)	83.6 ± 13.8 (49-113)(*P* = 0.67)	76.5 ± 13.8 (50-120)(*P* = 0.01)
Mean blood pressure (mmHg)	86.6 ± 14.1 (65-123)	91.3 ± 12.6 (67-120)(*P* = 0.13)	100 ± 13.6 (64-126)(*P* = 0.001)	92.2 ± 10.6 (66-114)(*P* = 0.06)

## DISCUSSION

We observed a significant increase in cuff pressure of tracheal tube (168% of the baseline values) after application of cervical retractors during anterior cervical discectomy. In few patients the cuff pressure increased to as high as 48 mmHg from baseline values of 10-12 mmHg. The mean and peak airway pressures also increased significantly [Table 1]. During ACDF surgery, retractor is placed in soft tissue of neck for better visualization of anterior spinal column. Cervical retraction increases tracheal cuff pressure thereby causing ischaemic injury to the tracheal mucosa, which may be responsible for occurrence of postoperative sore throat, dysphagia, and hoarseness. In a similar study, Kim and colleagues observed that during cervical retraction the cuff pressure of tracheal tube increased from a baseline of 20–32 mmHg.[1] Apfelbaum and colleagues in a cadaveric study demonstrated increased cuff pressure from 15–52 mmHg, after placement of retractor.[2] They observed that fixed tracheal tube with inflated cuff and tape at the mouth could exert asymmetric pressure on the trachea after the retractor placement, and possibly cause pressure related damage to recurrent laryngeal nerve.[2,3] Sperry *et al*. found that the mean cuff pressure of tracheal tube became 42.4 ± 7.0 mmHg with cuff inflation following intubation. The cuff pressure was adjusted to a ‘just seal’ pressure of 15.2 ± 1.6 mmHg.[4] However, it increased up to 43.2 ± 5.0 mmHg after traction and distraction.

Nitrous oxide (N_2_O) is known to diffuse into cuff of tracheal tube, increasing cuff pressure during intra-operative period.[5,6] In our study, the increase in cuff pressure was primarily related to retractor application as N_2_O was not used. Use of N_2_O could have caused further increase in cuff pressure. The initial cuff pressure may be inappropriately high in most of the patients, when the cuff is filled with recommended volume of air, and then, checking leakage with conventional techniques. We inflated cuff with minimal volume of air to achieve ‘just seal’ of the tracheal lumen.

Increased incidence of tracheal morbidity is related to longer duration of intubation and higher cuff pressure during retractor placement.[7] Complications like dysphonia and dysphagia are reported to persist even beyond 5 years after ACDF.[8] Laryngeal complications after ACDF include vocal cord paralysis secondary to recurrent laryngeal nerve injury.[3,9] Temporary unilateral vocal cord paralysis has been reported in the range of 0.98–8%. The incidence of permanent paralysis has been observed to be in between 0.15 and 3.5%.[9] These complications occur as a result of direct surgical trauma, nerve division, pressure or stretch induced neuropraxia, and ETT-related vocal fold palsy.[3,10] Audu and colleagues[11] reported 3.2% incidence of vocal cord palsy after ACDF which is comparable to the results of our study (2.7%).

Endotracheal intubation has been implicated for laryngeal morbidities such as vocal cord palsy with reported incidence of 15–94%.[12] Although the exact pathophysiology of post-intubation airway complications is not known, mucosal damage due to increase cuff pressure is thought to be responsible. Inflated cuff may impinge upon the nerve thereby pushing it against the thyroid lamina.[9] The cuff pressure is recommended to be kept within 20–30 cm H_2_O to provide adequate seal without compromising mucosal perfusion.[13] Capillary pressures of trachea decrease when the cuff exerts pressure greater than 30 cm H_2_O causing tracheal ischaemia. The severity of ischaemia is proportional to the pressure exerted by the cuff and the time of exposure.[12] Hence, maintenance of cuff pressure below tracheal mucosal capillary pressure is recommended, but the clinical evidence of efficacy of this practice in preventing tracheal morbidity is lacking. To prevent hyper-insufflation of the tracheal tube cuff, insufflations should be slow, with small volumes of air being added until a ”sealing“ pressure is reached, with no leaks during inspiratory phase of ventilation.[12]

It is reported that the intraoperative increase in cuff pressure and diminished electromyographic activity is associated with higher incidences of postoperative hoarseness.[9] These data further support the role of retractor/ETT interactions in vocal cord paralysis after ACDF.[9] Intraoperative recurrent laryngeal nerve monitoring for preventing its injury is one of the options but, not always feasible.

Normal venous and lymphatic pressures of trachea are 12 mmHg (16 cm H_2_O) and 3–5 mmHg; respectively.[14] Hence, cuff pressure maintained below 30 cm H_2_O may still affect these pressures. Cuff pressures above these values may provoke congestion and oedema of the tracheal mucosa. Adjustment of cuff pressure to an allowable limit after application of retractors may help prevent tracheal morbidity, but it has major limitations.[15] Pressure must be sufficient enough to seal the trachea to prevent aspiration of gastric contents and air leak during ventilation, simultaneously allowing adequate perfusion of tracheal mucosa. During retractor application, even though the cuff pressure increases, it is not advisable to aspirate air from the cuff as with readjustment of retractor, the optimum sealing may get disturbed.

There was increase in peak airway pressure in this study, though it was clinically not significant. It was possibly due to compression of the tracheal tube from surrounding tissue.

To conclude, the cuff and airway pressures of tracheal tube were increased significantly, after application of neck retractor during anterior cervical discectomy. The patients who developed postoperative tracheal injuries had increased cuff pressure. N_2_O, which could further increased the cuff pressure, was not used in these patients. Therefore, the cuff should be air-inflated to achieve ‘just seal’, with adequate cuff pressure monitoring. Nevertheless, the role of intermittent release of cervical traction, in prevention of tracheal ischaemia, may not be overemphasized. This study is limited by a small sample size. Hence, the effects of parameters like duration of surgery and volume of air required to seal the cuff, on cuff pressure could not be compared. Probably, a larger prospective randomized study would be required to validate these findings.
